# The 10th Santorini conference: Systems medicine, personalised health and therapy. “The odyssey from hope to practice: Patient first. *Keep Ithaca always in your mind*”, Santorini, Greece, 23–26 May 2022

**DOI:** 10.3389/fgene.2023.1171131

**Published:** 2023-03-09

**Authors:** Sophie Visvikis-Siest, Maria G. Stathopoulou, Raute Sunder-Plassmann, Behrooz Z. Alizadeh, Robert Barouki, Ekaterina Chatzaki, Georges Dagher, George Dedoussis, Panagiotis Deloukas, Alexander Haliassos, Brigitte Boisson Hiegel, Vangelis Manolopoulos, Christine Masson, Guillaume Paré, Markus Paulmichl, Alexandros M. Petrelis, Csilla Sipeky, Belgin Süsleyici, Georges Weryha, Alex Chenchik, Paul Diehl, Robin E. Everts, Alexander Haushofer, John Lamont, Ruth Mercado, Heiko Meyer, Herna Munoz-Galeano, Helena Murray, Ferrier Nhat, Charity Nofziger, Wolfgang Schnitzel, Stavroula Kanoni

**Affiliations:** ^1^ EA_1122 IGE-PCV, Université de Lorraine, Nancy, France; ^2^ Team 10: Control of Gene Expression, INSERM U, Centre Méditerranéen de Médecine Moléculaire C3M, Nice, France; ^3^ Department of Laboratory Medicine, Medical University of Vienna, Vienna, Austria; ^4^ Unit of Personalized Medicine, Department of Epidemiology, University Medical Center Groningen, University of Groningen, Groningen, Netherland; ^5^ Université de Paris, Inserm unit 1124 (T3S), Paris, France; ^6^ Laboratory of Pharmacology, Medical School, Democritus University of Thrace, Alexandroupolis, Greece; ^7^ Institute of Agri-Food and Life Sciences, Hellenic Mediterranean University Research Centre, Heraklion, Crete, Greece; ^8^ Inserm, Paris, France; ^9^ Graz Medical University, Graz, Austria; ^10^ Milano-Bicocca University, Milan, Italy; ^11^ Beijing Academy of Sciences, Beijing, China; ^12^ Department of Nutrition and Dietetics, Harokopio University of Athens, Athens, Greece; ^13^ William Harvey Research Institute, Barts and the London School of Medicine and Dentistry, Queen Mary University of London, London, United Kingdom; ^14^ EurSpLM, ESEAP, The Greek Proficiency Testing Scheme for Clinical Laboratories Athens, Athens, Greece; ^15^ Clinical Pharmacology and Pharmacogenetics Unit, Academic General Hospital of Alexandroupolis, Alexandroupolis, Greece; ^16^ Population Health Research Institute, Genetic and Molecular Epidemiology Laboratory, McMaster University, Hamilton, ON, Canada; ^17^ Privatklinik Maria Hilf, Klagenfurt, Austria; ^18^ Digitsole, Nancy, France; ^19^ UCB Pharma, Translational Medicine, Precision Medicine and Biomarkers, Genetics, Braine-l’Alleud, Belgium; ^20^ Marmara University, Faculty of Sciences and Letters, Department of Molecular Biology, Istanbul, Türkiye; ^21^ Cellecta, Inc, Mountain View, CA, United States; ^22^ Agena Bioscience, San Diego, CA, United States; ^23^ Inst. f. Med. u. Chem. Labordiagnostik, Klinikum Wels-Grieskirchen GmbH, Wels, Austria; ^24^ Randox Laboratories Limited, Crumlin, Co. Antrim, United Kingdom; ^25^ Agena Bioscience, Hamburg, Germany; ^26^ HMG Systems Engineering GmbH, Fürth, Germany; ^27^ Thermo Fisher Scientific, San Francisco, CA, United States; ^28^ PharmGenetix GmbH, Anif/Niederalm, Austria

**Keywords:** systems medicine, personalized medicine, pharmacogenomics, medical diagnostic, cancer, heart inflammation, genetic screening, “-OMICs” biomarkers

## 1 The 10th Santorini conference

After a 2-year hiatus, due to the recent COVID-19 pandemic, the 10th biannual conference on Systems Medicine, Personalised Health and Therapy, under the auspices of the Santorini Conferences Association (SCs), the International Federation of Clinical Chemistry and Laboratory Medicine (IFCC), the European Federation of Clinical Chemistry and Laboratory Medicine (EFLM), the Hellenic Society of Pharmacogenomics and personalized Diagnosis and Therapy (EEPHARM), and the European Society of Pharmacogenomics and Personalised Therapy (ESPT), took place in Santorini, Greece, between 23 and 26 of May 2022.

It was sponsored by several companies: Randox (Crumlin, United Kingdom), Agena Bioscience (Hamburg, Germany) and Cellecta as **Gold Sponsors**; Thermofisher Scientific (San Francisco, United States) and PharmGenetix (Vienna, Austria), as **Silver Sponsors**; HMG systems Engineering (Fuerth, Germany) as **Bronze Sponsors**, reinforced by specific supports from the Santorini Conferences Association (SCs), The Austrian Society for Laboratory Medicine & Clinical Chemistry (OGLMKC), Transgene (Strasbourg, France), The Journal “Frontiers in Genetics” and strengthened with scientific meetings supported by the University of Lorraine and INSERM (“Cercle Gutenberg”).

For this 20th anniversary, we attracted many world-renowned delegates (researchers, scientists, biologists, pathologists, oncologists, genetic epidemiologists, pharmacogeneticists and biobanking experts, both from the academia and the industry) from 28 countries. The event was particularly successful and of high scientific quality. The 10th Santorini Conference, under the Presidency of Sofia Siest, the director of the EA_1122; IGE-PCV (http://ige-pcv.univ-lorraine.fr/en/), University of Lorraine, France, offered a diverse and innovative scientific program, showcasing the work of 35 worldwide distinguished speakers, who shared recent advances on personalized medicine with 127 attendees in eight distinct sessions.

The Conference was initiated by a keynote round table on “PGx analysis in the medical diagnostic laboratory–from science to clinical decision support (CDS)” and was followed by a keynote lecture on “Advances in Cancer detection”.

The **second day** was focused on: “Liquid Biopsy, Past, Present, Future”—“Advances on Cellular and Multi-Omic Approaches” and “Approaches for the Discovery of Drug Targets, Resistance Mechanisms and Biomarkers” and included a flash oral communication session.

The **third day** sessions were on “Heart inflammation”, “Econ-Omics: Better Care for Better Cost”, “Genetic Screening & Clinical Applications (part I and II.

The **fourth and** last day ended out with an oral communication session, a session on “Digital Health” and a closing presentation made by Sofia Siest.

In this article we briefly outline the presentations delivered during the conference and review the key messages and conclusions.

## 2 The odyssey from hope to practice

The conference was officially initiated by Sofia Siest, the President of the Santorini Conference series, thanked all the members of the different committees and the sponsors. She presented an overview of the outcomes of the previous conference and announced the inauguration of the Santorini Conferences series under the umbrella of the Santorini Conferences Association (SCs) and the development of the new website: www.santoriniconference.org. She then underlined that this was the 20th Anniversary of the conference’s series and outlined the content of the scientific program (presentations and speakers) and the different topics to be covered.

Following the welcome session, **Raute Sunder-Plassmann** (Vienna, Austria) and **Markus Paulmichl** (Salzburg, Austria) introduced the keynote round table, supported by the

“OGLMKC” and focused on Pharmacogenetics (PGx) Analysis in the Medical Diagnostic Laboratory–From Science to Clinical Decision Support (CDS).

The major topics/highlights of the round table discussion included.

• Whole genome sequencing provides opportunities to identify new genetic factors for efficacy and safety phenotypes or for explaining the missing phenotype heritability seen in twin studies. New genetic variants could act as regulators of pharmacogenes expression.
• Long read sequencing allows to unravel complex gene loci.
• Large biobanks offer the opportunity to discover pharmacogenomic phenotypes.
• Value in expanding pharmacogenomic research to diverse ancestry groups.
• Significance of personalized prescription.
• Advances in genotyping technologies and a concomitant drop in the costs and turnaround time facilitate multi-gene analyses and preemptive pharmacogenetic testing in medical diagnostic laboratories.
• Value in switching to extended PGx panel diagnostics.
• Adhere to current guidelines on pharmacogenomic testing and reporting.
• Importance of recommendations for standardization of pharmacogenetic terminology and test design.
• Importance of providing clear, concise, and interpretable reports, providing details on the test itself and the identified variants in a separate supplementary document.
• Need for integrating PGx data into the patient’s electronic health record and—if available—in a Clinical Decision Support System (CDSS).

**M. Ingelmann Sundberg** (Stockholm, Sweden) described how the ability to interrogate the whole genome provides us with unparalleled opportunities to identify new genetic predisposing factors in an unbiased manner for both efficacy and safety phenotypes, often leading to new insights into gene function and explaining the missing heritability seen in twin studies. Rare variants, which are unaccounted for during standard genotyping, actually explain up to 4%–6% of the variability in certain genes encoding enzymes and transporters. Similarly, haplotypes in linkage equilibrium to variants defining a specific CYP allele may cause altered CYP activity due to additional variants affecting gene expression. Additionally, variants in genes not directly linked to the *ADME* gene in question may influence its regulation, as recently demonstrated for the nuclear factor 1B (NFIB)- dependent regulation of *CYP2D6* expression and risperidone metabolism in psychiatric patients. Long read sequencing technologies allow to unravel complex gene loci such as the *CYD2D*locus.

**M. Pirmohammed** (Liverpool, United Kingdom) explained how the availability of large-scale biobanks, such as the United Kingdom biobank, enables us to identify novel pharmacogenomic phenotypes—but may be limited by the depth of clinical phenotypes within the biobanks. Additionally, it is important to expand pharmacogenomic research to different ethnic groups to ensure that what we discover and implement is relevant to all population and that we do not exacerbate race and health inequalities.

**Ron HN van Schaik** (Rotterdam, Netherlands) presented how selected centers reported good results for improving medication safety and efficacy by including common genetic variations in genes encoding enzymes, transporters and targets into pharmacogenetic guided treatment decisions. However, comprehensive and/or preemptive pharmacogenetic analyses and their implementation into daily clinical practice is still not very common. Physicians usually rely on reactively ordered PGx tests for selected variants in a single or only a few genes and particularly on a short turnaround time to initiate, adjust or change a standard therapeutic regime. Due to recent advances in genotyping technologies and a concomitant drop in the costs and turnaround time for multi-gene analyses, PGx panel diagnostics using single-nucleotide polymorphism (SNP) genotyping arrays or next-generation sequencing (NGS) based approaches are established in a few medical diagnostic laboratories. But further efforts of wider implementation to the entire healthcare systems is needed. There is also a patient driven demand for PGx guided treatment decisions and multigene/multivariant testing. Preemptive genotyping would ensure that the appropriate genetic information to guide drug therapy is already available when needed and will maximize the effect of PGx.

**R. Sunder-Plassmann** (Vienna, Austria) described how the availability of an abundance of PGx data still represents a huge challenge for physicians, who frequently struggle with the interpretation and application of PGx test results. Hence, PGx reports have to be designed in such a way that the data is easy to comprehend, and the focus is on the current medication and relevant actionable genetic variants, preferentially in a separate short report for immediate use. In addition, a comprehensive report explaining the patient’s pharmacogenetic profile in more detail should be also provided. According to current recommendations (ACMG), the patient’s genotype, predicted PGx-phenotypes, commonly used drugs that may be affected by the identified genotype, a statement that alternative medication might be considered (if applicable) and resources for guidelines should be reported. Additionally, they suggest reminding physicians that the accuracy of the anticipated PGx phenotype is dependent on the variants identified and that co-medication and drug-drug interactions may also influence the phenotype.

For future therapeutic decisions, PGx data should be included in the patients’ electronic health records. Currently, only few *in vitro* diagnostic tests are commercially available for PGx analyses. The majority of medical diagnostic laboratories that offer PGx panel diagnostics use lab developed assays, which may vary in gene selection, variants detected, and nomenclature for phenotype description. This may lead to discrepancies in the test results between laboratories and the need for standardizing the minimal requirements for a diagnostic PGx test.

**Markus Paulmichl** (Salzburg, Austria) closed the session with a presentation on “Standardization in PGx diagnostics”.

George Dagher (Paris, France) and Sofia Siest (Nancy, France) introduced the keynote lecture on **“Advances in Cancer Detection”** presented by **Nickolas Papadopoulos** (Baltimore, United States). Nickolas Papadopoulos highlighted that the earlier a cancer is detected the higher the chance for a successful outcome. For many cancers there are not any screening modalities available. The ability to identify cancers through blood testing is one of the most exciting advances in cancer diagnostics. In a screening setting it provides the opportunity to detect multiple cancer types with a single test. Liquid biopsy also has the potential to detect early signs of minimal residual disease and recurrence. The presentation outlined these opportunities along with challenges associated with such clinical applications. We discussed the biomarkers, technologies and the type of studies required to develop and evaluate the utility of such tests, in the first session of the next conference day.

## 3 Conference sessions

### 3.1 Session 1: Liquid BIOPSY, past, Present, future

The session was chaired by Georges Weryha, Nancy, France, and Georges Dagher, Paris, France, and sponsored by AGENA.

**Klaus Pantel** (Hamburg, Germany) started the session with a talk on “Liquid Biopsy: From Discovery to Clinical Implementation”, highlighting the utility of liquid biopsy in clinical practice. The molecular analysis of circulating cell-free tumour DNA (ctDNA) and circulating tumour cells (CTCs) released into the blood can provide clinically relevant information as “liquid biopsy” (Pantel and Alix-Panabieres, 2019; Alix-Panabieres and Pantel, 2021) and provide new insights into tumour biology (Keller and Pantel, 2019). A variety of targeted and non-targeted approaches have been used to assess ctDNA, including NGS and MassARRAY-Based ctDNA assays (Belloum et al., 2020; Schneegans et al., 2020). Liquid biopsy analyses with validated platforms provide information on early detection of cancer, identification of cancer patients at risk to develop relapse (prognosis), and it may serve to monitor tumour evolution, therapeutic targets or mechanisms of resistance on metastatic cells. New promising liquid biopsy markers include extracellular vesicles, circulating microRNAs and tumour-educated platelets as well as circulating host cells. Technical standardization and clinical validation of liquid biopsy assays are essential (Connors et al., 2020).

**Catherine Alix-Panabières** (Montpellier, France), continued the session with a talk on the **“Metastasis-Competent Circulating Tumor Cells in Colon Cancer”**, elaborating on the need of developing a reliable, standardized and robust method to expand CTCs from different cancer types. Real-Time Liquid Biopsy has been introduced (Pantel and Alix-Panabieres, 2010) as a new diagnostic concept predicated on the analysis of circulating tumor cells (CTCs) or circulating tumor-derived factors, in particular, cell-free tumor DNA (ctDNA). Highly sensitive liquid biopsy assays have been developed that can now be applied to detect and characterize minimal residual disease (Pantel and Alix-Panabieres, 2019; Alix-Panabieres and Pantel, 2021). Furthermore, CTCs are promising new biomarkers for prognostic prediction and monitoring of therapies in patients with solid tumors, as well as understanding the biology of metastasis in cancer patients (Alix-Panabieres and Pantel, 2014). However, an in-depth investigation of CTCs is hampered by the very low number of these cells, especially in the blood of colorectal cancer patients. Thus, the establishment of cell cultures and permanent cell lines from CTCs has become the most challenging task over the past year (Cortes-Hernandez et al., 2020). Alix-Panabieres et al. described, in 2015, the *in vitro* expansion of colon CTCs and established the first permanent cell line from CTCs of a metastatic colon cancer patient (Cayrefourcq et al., 2015). This colon CTC line designated CTC-MCC-41 is in culture for more than 6 years and has been characterized at the genome, transcriptome, proteome and secretome levels. This thorough analysis showed that CTC-MCC-41 cells resemble characteristics of the original tumor cells in the colon cancer patient and display a stable phenotype. The molecular portrait of CTC-MCC-41 line displays a very specific transcription program completely different than those of the primary and metastatic colon cancer cell lines (Alix-Panabieres et al., 2017). More recently, Alix-Panabieres’ team characterized eight additional CTC lines using blood samples from the same metastatic cancer: a unique biological material collected before and after chemotherapy and targeted therapy, and during cancer progression (Soler et al., 2018). More recently, they showed that the PI3K/AKT/mTOR signaling pathway plays a key role in the proliferation of the CTC-MCC-41 line (Smit et al., 2020) and that the selective treatment pressure in colon cancer drives the molecular profile of resistant CTC clones (Cayrefourcq et al., 2021). Although, viable CTCs are not exploitable in all patients with cancer, they are a precious tool to unravel the mechanisms of metastasis formation and cancer cell dissemination through the identification/characterization of the aggressive tumor cells that need to be eradicated. We are progressing very fast in the field of *liquid biopsy* in cancer research. However, much effort should now focus on developing a reliable, standardized and robust method to expand CTCs from different cancer types (Alix-Panabieres, 2020). The establishment of CTC lines represents a new opportunity to decipher the metastatic cascade and, hopefully, to find ways to stop cancer dissemination.

**Ed Schuuring** (Groningen, Netherlands)**,** took the floor and gave a talk on the **“Detection of clinically actionable mutations in NSCLC: is there a one-fits-all cell-free DNA test for routine clinical practice?”**. Circulating tumor DNA (ctDNA) is a potential minimally invasive molecular tool to guide treatment decision making and disease monitoring especially when no appropriate tumor tissue biopsy is available. A suitable diagnostic-grade platform is required for the detection of tumor-specific mutations in circulating cell-free DNA (ccfDNA) with high sensitivity. Dr Schuuring described the objective of their study to investigate if a one-fits-all ccfDNA test exists for the different applications in the molecular diagnostics of lung cancer such as molecular profiling for treatment-decision-making, response monitoring and detection of treatment-resistant mechanisms. The design of the study was to determine the concordance between various ccfDNA tests using plasma collected at various time-points during therapy from NSCLC patients treated with tyrosine-kinase and immune checkpoint inhibitors. The researchers evaluated the Roche Cobas^®^ EGFR Mutation Test v2, the Agena UltraSEEK^®^ Lung Panel, tumor-mutation-specific Bio-Rad®-ddPCR assays and the Roche AVENIO ctDNA Expanded NGS Kit. Based on the results presented, the concordance to detect clinically relevant mutations in plasma comparing the different ccfDNA tests was >90% for those mutations covered by the different assays. The concordance between therapeutically targetable mutations detected in tumor tissue with NGS and in the pre-treatment plasma samples was high for all assays (∼80%) and in agreement with reported data. In conclusion, there seems to be no one-fits-all ccfDNA test for all clinical application. To select the appropriate ccfDNA test for clinical questions that lead to actionable mutations, aspects like the complexity of the test, costs, reimbursement issues, turn-around-time, the number of relevant mutations covered by each assay, expertise of the diagnostic lab and availability of ccfDNA, are important. Several studies presented were supported by the CANCER-ID consortium (including Roche and Agena Bioscience) and unrestricted research grants of Bristol Myers Squibb, Bio-Rad, Biocartis, and Agena Bioscience. Various studies were based on collaborations with MUG Graz (E Heitzer), Imagenome Montpellier (P-J Lamy) and UKE Hamburg (H Wikman).

The session was concluded by the talk from Oellerich et al. (Göttingen, Germany), on “Donor-derived cell-free DNA testing in organ transplantation: a value proposition”. Dr Oellerich outlined a value proposition for donor-derived cell-free DNA (dd-cfDNA) testing in organ transplantation. There is a need to improve personalized immunosuppression in organ transplantation to reduce premature graft loss. Biomarkers are needed to better detect rejection, asymptomatic graft injury, and under-immunosuppression. Assessment of minimal necessary exposure to guide tapering and prevent immune activation is also important. There is robust clinical evidence from more than 50 published studies supporting the role of dd-cfDNA for monitoring graft integrity and detection or exclusion of rejection. The value proposition for the patient includes earlier transplant injury intervention, less full blown rejection risk, an alternative to invasive biopsies, personalized immunosuppression with potential for improved long-term outcome. Transplant physicians benefit from better immunosuppressive guidance and having an alternative when biopsies are refused or contraindicated. Further advantages are improved biopsy interpretation, less trial and error changes in immunosuppression, and less time dealing with complications. The laboratory medicine specialist can provide more effective services. Hospital management and insurance companies could benefit from more cost-effective surveillance of transplant recipients. Potential cost savings would be due to fewer biopsies as a result of the high negative predictive value, fewer retransplantations, or less organ failure with return to dialysis. A pathway to implementation and metrics was suggested to measure the effectiveness of dd-cfDNA testing.

### 3.2 Flash Communications session (supplement)

**The session was chaired by Stavroula Kanoni, London, United Kingdom and Vangelis Manolopoulos**, **Alexandroupolis, Greece**

### 3.3 Session II–advances on cellular and multi-omic approaches

The session was chaired by Behrooz Z. Alizadeh, Groningen, Netherlands and Georges Weryha, Nancy, France

The first talk of this session was from **Colin J.H. Brenan** (Boston, United States), presenting the **“Engineering a Rational Approach to Precision Oncology Dugs”**. Clinical trials of new oncology drugs have a staggering 97% failure rate typically due to toxicity or lack of drug efficacy. One common issue is failure to understand the mechanism of action and misidentification of putative biomarkers indicative of drug response. To address this issue, Dr Brenan and his team developed and clinically deployed an innovative implantable microdevice (called the Nanonail™) for functional and simultaneous *intra tumor* molecular profiling of tumor sensitivity to up to 18 different oncology drugs and/or drug combinations per microdevice at the single cell level and with the tumor in its native microenvironment. Each unique drug or drug combination was loaded into one of the micromachined depots of the Nanonail for delivery of a controlled dose of drug to the surrounding tumor tissue once inserted into the tumor with a standard fine needle biopsy tool. After 1–3 days the device was recovered with a plug of surrounding tissue and processed according to standard histopathology protocols. Thin sections of the zone of drug-tumor interaction were individually analyzed to create a detailed, high resolution spatial multi-omic profile of the tumor response to each agent. An additional benefit, molecular profiling the tumor-drug response along the spatial diffusion profile of drug from each depot provides key information on tumor response to different drug doses. Dr Brenan provided examples on how application of single cell functional profiling can reveal novel, potent anti-tumor drug combinations and in particular the combination of an immunotherapy with molecularly targeted or cytotoxic agents.

The second presenter of this session was Ekaterini Chatzaki (Alexandroupoli, Greece), describing her recent work on “Biomarker discovery in the era of automated machine learning: from targeted to data-driven approaches”. Biomarkers are the cornerstone of precision medicine: identified as a measurable indicator of some biological state or condition, they promise to offer solutions for accurate diagnosis, prognosis and therapeutic monitoring. Dr Chatzaki and her team have been studying methylation in liquid biopsy material in different pathological conditions such as cancer and diabetes. The team has moved gradually from hypothesis-driven to (big) data-driven approaches, as modern -omics technologies lead the accumulation of large precious multi-parametric biological datasets. They employed *ad hoc* auto machine-learningtools for data extrapolation, delivering low-feature validated models/classifiers and suggest that this approach can have unprecedented added value in different medical conditions.

### 3.4 Session III–approaches for the discovery of drug targets, resistance mechanisms, and biomarkers

The session was chaired by Stavroula Kanoni, London, United Kingdom and Behrooz Z. Alizadeh, Groningen, Netherlands and sponsored by CELLECTA

**Paul Diehl** (Cellecta, Inc, Mountain View, California, United States) presented “**Flexible and Scalable Genetic Screens for Discovery and Characterization of Novel Therapeutic Targets”**. The measurements of changes in gene activation and expression provide a basis to understand the genetic changes that cause biological responses of interest. Cell-to-cell gene disruption induced by CRISPR and other gene-perturbation technologies help tease out the drivers required for these responses. Dr Diehl discussed how adaptations of these two screening approaches can be used to discover the genetic drivers responsible for phenotypic variabilities, such as drug sensitivities, disease variation, and degrees of differentiation within cell populations, across tissue microenvironments, and between single cells. Also from the same company, **Alex Chenchik** talked about **“Immunophenotyping of T Cell receptor and B Cell receptor clonotypes”**. T Cell receptor (TCR)/B Cell receptor (BCR) repertoire profiling holds great potential for understanding disease mechanisms. Dr Chenchik explained how they introduced a novel technology for profiling of all human TCR and BCR variable regions and phenotypic characterization of immune cells in bulk and at the single-cell level in PBMCs and immune cell fractions. Preliminary data showed that TCR/BCR clonotype analysis combined with targeted expression profiling of immune cells can be applied for large-scale discovery in several immune-responsive model systems.

### 3.5 Session IV–heart inflammation

The session was chaired by Panagiotis Deloukas, London, United Kingdom and Georges Weryha, Nancy, France and sponsored by RANDOX

**Federica Marelli-Berg** (London, United Kingdom) gave a talk on “Trac**king T cell-mediated autoimmunity in the heart”.** Autoimmune cardiac inflammation is becoming recognized as a key contributing factor in heart muscle diseases. Despite advances, the functional features of cardiac immunity in humans remain largely undefined, due to the technical challenges of studying the immune response *in-situ*. Dr Marelli-Berg and others described a population of cardiotropic T Cells (cT-cells) characterized by the expression of the hepatocyte growth factor receptor cMet and the chemokine receptors CCR4 and CXCR3. They showed that memory, activated cT-cells significantly increase in the circulation and in the heart of patients with inflammatory cardiomyopathies, but not in acute myocardial infarction or healthy controls. cT-cells divide preferentially in response to the autoantigen cardiac myosin and display similar functional features in acute and chronic cardiac inflammation. In experimental autoimmune myocarditis, which recapitulates the autoimmune phase of human myocarditis, development of cT-cells and disease can be prevented by pharmacological cMet inhibition, suggesting a causative role for this T Cell subset.

Next, **Behrooz Z. Alizadeh** (Groningen, Netherlands) described the **“Predictive value of Inflammatory causes of vascular disorders in Personalized medicine”.** The pivotal role of inflammation in cardiovascular diseases (CVD) has been scrutinized for a century. Accumulating number of studies suggest the involvement of specific molecular pathways in the disease mechanism, which are represented by inflammatory biomarkers and are claimed potential targets for therapeutics (NFκB, OPN). However, there is little known whether these associations are causal and are dependent on the dominant type of inflammatory cells. Dr Alizadeh presented the latest results of their studies on the causal association of inflammatory biomarkers with major CVD phenotypes, by using genetic risk. They also evaluated the potential application of inflammatory biomarkers in better prediction of disease outcomes. Future investigations should focus on the crosstalk between causal inflammatory biomarkers, the type of inflammatory cell involved, in the pathological contexts of cardiac cells and may eventually lead to specific inflammatory-based therapies for the personalized prevention and treatment of CVD.

The last talk in this session was given by **Helena Murray** (Randox Laboratories Limited, Crumlin, Co. Antrim, United Kingdom), discussing the **“Development of a Type I Diabetes Genetic Risk Array”.** Differentiating between Type 1 diabetes (T1D) and Type 2 diabetes (T2D) is challenging due to the increasing incidence of childhood obesity blurring the traditional T1D *versus* T2D timelines. More young people are getting T2D and T1D can occur at any stage in life and an increasing number of cases of T1D is also occurring at old age. Currently available diagnostic tests have several limitations in accurately diagnosing diabetes subtypes with up to 15% of young adults wrongly classified and treated. The aim of this study was to consider genetic predisposition as an aid to improve diabetes classification. Genetic predisposition to diabetes is largely determined by the presence of human leukocyte antigen (HLA) genes. Genome-wide association studies have identified additional non-HLA SNPs, robustly linked with T1D. Combining these, a 10 SNP genetic risk score (GRS) was developed which can aid discrimination between T1D and T2D, particularly when used in conjunction with clinical features and autoimmune markers. The assay employs multiplex Polymerase Chain Reaction (PCR) coupled to Biochip Array Technology (BAT, Randox Laboratories Ltd., Crumlin, UK) to genotype 10 SNPs associated with T1D (Oram et al., 2016). Assay optimization and specificity was achieved using pre-characterized DNA samples and initially validated by testing DNA samples (n = 259) provided by University of Exeter. The T1D GRS array is capable of rapidly detecting all 10 SNPs associated with T1D. Through an associated algorithm, the array can generate a T1D Genetic Risk Score, which in conjunction with conventional methods, can distinguish T1D from other subtypes. This assay has potential to prevent misdiagnosis of diabetes and facilitate improved patient management.

### 3.6 Session V–econ-omics: Better care for better cost

The session was chaired by Georges Dagher, Paris, France and Belgin Süsleyici, Istanbul, Turkey

The first talk in this session was given by one of the chairs, **George Dagher** (Paris, France), on **“Big data, Artificial Intelligence and ethics”.** Big data is certainly an essential component of digital science and technology and also of machine learning, robotics, and new means of communication. The information that the data initially contains, is considerably enriched by cross-referencing data. Highly diverse, this data can be related to health or wellbeing. One of the characteristics of big data in health is the blurring of the distinctions underpinning implementation of the ethical principles that promote the protection of individual rights in health. Precise knowledge of individuals and of their state of health creates a risk of profiling, which threatens the protection of private life and may lead to stigmatization of people or groups. Such stigmatization threatens private life, but also the principles of solidarity and equity which are the basis of our health system. *Care* and *business* are becoming increasingly hard to distinguish, as a result of the transformation of care and of the healthcare market*.* The need for protection of the individual must be reaffirmed and its modalities redefined, to dispel the threat of a society under the surveillance and control of multiple providers acting for various purposes.

The second talk in this session was given by **Uwe Oelmueller** (QIAGEN GmbH, Hilden, Germany) on **“Standardized Preanalytics: The Key for Reliable Diagnostics, Research and Biobanking”**. Molecular *in vitro* diagnostics and research have allowed great progress in medicine including diagnostics. However, profiles of these molecules (nucleic acids, proteins, and metabolites) can change significantly during specimen collection, transport, storage, and processing. This can make the outcome from diagnostics or research unreliable or even impossible. High quality specimens with preserved analyte profiles are crucial for reliable diagnostics, biomedical research and biobanking. Specifying, developing and verifying pre-analytical workflow parameters for diagnostics tests has consequently become a requirement by new European legislation. The EU SPIDIA Consortium (2008–2013) developed new pre-analytical technologies for preserving molecular profiles in human specimen and generated broad evidence that guidance to laboratories on pre-analytical workflows improves analytical test results. Based on these results, the CEN/TC 140 for “*in-vitro* diagnostic medical devices” had released first 9 European Technical Specifications for pre-analytical workflows addressing different blood, other body fluids and tissue based molecular applications. In 2018 and 2019 they progressed to International Standards at the ISO/TC 212 for “clinical laboratory testing and *in vitro* diagnostic test systems”. The successor EU SPIDIA4P consortium project (2017–2021), supported by a large international network, has broadened to a final portfolio of 22 pre-analytical CEN and ISO Standards intending to improve *in vitro* diagnostics and biomedical research, has developed corresponding External Quality Assurance (EQA) and is driving international implementation. The SPIDIA project received funding from the EU’s FP7 under grant agreement no. 222916. The SPIDIA4P project received funding from the EU’s Horizon 2020 research and innovation program under grant agreement no. 733112.

### 3.7 Session VI—first part–genetic screening and clinical applications

The session was chaired by **Guillaume Paré, Hamilton Canada** and **George Dedoussis, Athens, Greece**

**Georges V Dedoussis** (Athens, Greece) presented recent work on the **“Omics and Mastiha treatment in NAFLD - The EU Mast4Health program”**. Non-alcoholic fatty liver disease (NAFLD) is a major public health concern in both industrialized and developing nations, with an estimated global incidence of 25% in the general population and with limited treatment approaches. The Mast4Health consortium investigated the effect of the nutraceutical Mastiha supplement in the omics profile of patients with NAFLD, within a multicenter, randomized, double-blinded, and placebo-controlled clinical trial design. Based on the results, there was an improvement in liver inflammation and fibrosis (as assessed by MRI and the use of the sensitive LiverMultiScan software). Post-treatment levels of both Liver Inflammation Fibrosis score (LIF) and iron-corrected T1 (cT1) were lower in the Mastiha group compared to the Placebo among volunteers with BMI>35 kg/m^2^. The Bray-Curtis dissimilarity index between baseline and post-treatment bacterial communities was larger in the Mastiha group *versus* the Placebo. The metabolomic analysis showed a significant reduction of Lysophosphatidylcholines and Lysophosphatidylethanolamines in the Mastiha group suggesting that Mastiha exhibits a beneficial effect in phospholipid homeostasis. In conclusion, after 6 months of Mastiha supplementation, the investigators observed a significant improvement on microbiota dysbiosis and lipid metabolite levels in patients with NAFLD (Amerikanou et al., 2021). This project received funding from the European Union’s Horizon 2020 research and innovation program MAST4HEALTH under the Marie Skłodowska-Curie grant agreement no 691042. (NCT03135873, www.clinicaltrials.gov).

### 3.8 session VI—second part–genetic screening and clinical applications

The session was chaired by **Guillaume Paré (Hamilton Canada)** and **Csilla Sipeky (Brussels, Belgium**).

The first speaker in this session was **Panagiotis Deloukas** (London, United Kingdom) discussing **“Polygenic Risk Scores, application and challenges in cardiovascular disease prediction”.** A total of 14% of adults above the age of 16 have doctor-diagnosed cardiovascular disease (CVD) which is a leading cause of death (∼25% of all deaths) and disability in the United Kingdom. Coronary Heart Disease (CHD) is the leading cause of CVD death in both sexes (14% men, 8% women). South Asians have a 2-fold higher risk for CHD compared to European-descent individuals and an earlier onset of disease. A polygenic risk score (PRS) aggregates all known genetic variants associated to the disease. Dr Deloukas and others developed a European CHD-PRS using the data from the latest CARDIoGRAMplusC4D meta-analysis in 1,165,690 participants including 181,522 cases (2.3 M genetic markers) and evaluated this new CHD-PRS in the Malmo Diet and Cancer study, confirming its predictive value and ability to predict secondary cardiovascular events. LDL-cholesterol is a major CHD risk factor. In a parallel study of LDL-cholesterol in 1.65 M individuals, the team showed that the multi-ancestry PRS had the best or near-best performance in each ethnic group tested, with improved or equivalent prediction relative to ancestry-matched scores (Graham et al., 2021). The team is further developing a multi-ancestry CHD PRS and aim to validate its performance in 49,000 British South Asians. Whilst pursuing investigation of disease prevention in the British population, the team is also assessing the cost-effectiveness and clinical value of the CHD-PRS in improving management of those with established disease. As part of a national UK effort (Our Future Health program) the aim is to look at disease severity and onset in patients with higher PRS, including rare CVD.

The next speaker, **Robert Barouki (**Paris, France) gave a talk on “**The relevance of non-genomic stressors: deciphering environmental factors for the next decade”**. Climate change, urbanisation, chemical pollution, and disruption of ecosystems, including biodiversity loss, affect our health and wellbeing. HERA is an EC-funded H2020 CSA project aiming at providing a research agenda for the next 10 years in the field of environment climate and health (https://www.heraresearcheu.eu/). The agenda identifies six major research goals in these fields. These include research to 1) reduce the effects of climate change and biodiversity loss on health and environment, 2) promote healthy lives in cities and communities, 3) eliminate harmful chemical and physical exposures, 4) improve health impact assessment and implementation research, 5) develop infrastructures, technologies and human resources and 6) promote research on transformational change towards sustainability. Numerous specific recommendations for research topics are presented under each research goal. The results call for an unprecedented effort to support a better understanding of the causes, interlinkages and impacts of environmental stressors on health and the environment. This will require breakdown of silos within policies, research, actors as well as in our institutional arrangements in order to enable more holistic approaches and solutions to emerge.

Next in the session, **Guillaume Paré** (Hamilton, Ontario, Canada) presented his recent work on **“Digging deep for translational gold: Multi-omics approach to cardio-metabolic traits”**. Despite recent advances in acute diagnosis and treatment of cardio-metabolic diseases (CMD), the development of new blood biomarkers for risk stratification has been slow. The majority of reported biomarker-disease associations fail to enter clinical practice due to their inabilities to discriminate risk, or more importantly, due to a lack of evidence that they represent causal associations with risk of disease. Distinguishing modifiable, causal mediators from the many biomarkers that are statistically linked to CMDs is a primary challenge in molecular epidemiology. Truly causal biomarkers such as LDL cholesterol have been invaluable in the prevention, treatment and identification of at-risk individuals. Dr Paré proposed an integrated genomic-proteomic (biomarker) approach to identify novel causal mediators of CMDs, and illustrated with examples from coronary artery disease, stroke, diabetes, chronic kidney disease and obesity. This approach is based on Mendelian Randomization (MR) that protects against confounding and reverse causation. Integrating genetics and high-throughput proteomics holds the promise of better risk stratification, identification of new disease pathways, and paves the way for novel therapeutic interventions.

Next in this session, **Stavroula Kanoni** (London, United Kingdom) gave an overview of recent developments around the **“Genetic susceptibility for COVID-19 infection and severity”**. The COVID-19 pandemic, caused by infection with SARS-CoV-2, has led to a total of 373 M cases worldwide and 5.5 M deaths. The SARS-CoV-2 infection has varied consequences, ranging from asymptomatic, to mild flu-like symptoms, to life-threating consequences like viral pneumonia and acute respiratory distress syndromes. Risk factors associated with the disease severity include increasing age, male gender, other comorbidities, and ethnicity. Furthermore, host genetic factors have also been identified as risk factors of SARS-CoV-2 infection or severe consequences of COVID-19, through genome-wide association studies (GWAS), whole-genome sequencing (WES) and candidate gene studies. Genes implicated with infection susceptibility or disease severity are involved in key pathophysiological processes, including viral entry into cells, immunity, and inflammatory responses. The most putative causal COVID-19 genes include *SLC6A20*, *ABO*, *CXCR6*, *INFAR2*, *OAS* (1,2,3), *DPP9*, *TYK2*, *ACE*, *MUC5B* and *FOXP4* and are linked to increased susceptibility and/or severity. Genetic predisposition to severe COVID-19 is also associated with deep venous thrombosis, morbid obesity, renal failure, pulmonary heart disease and respiratory failure. Large scale observational and Mendelian randomization studies have identified smoking as a risk factor for infection and severity, while there is no protective effect of vitamin D on COVID-19 susceptibility, severity, or hospitalization. The development of vaccines against SARS-CoV-2 have proven very efficient at halting the spread. The emergence of mutated variants of the virus are causing concern but vaccine engineering could be implemented to match the need of a response to the new variants. It is very crucial to increase the genomic surveillance around the world and at the same time increase the vaccine uptake.

### 3.9 Selected abstracts—oral communications session (supplement)

Session chaired by **Stavroula Kanoni, London, United Kingdom** and **Vesna Dimitrejevic Sreckovic, Belgrade, Serbia**

### 3.10 session VII–digital health

The session was chaired by Alexander Haliassos, Athens, Greece and Ekaterini Chatzaki, Alexandroupolis, Greece

Alexander Haliassos (Athens, Greece) presented recent advances in “Mobile Health (mHealth) and Internet of Things (IoT)”. When internet-based enabling technologies are coupled with new capabilities in mobile communications providing remote access from everywhere using web enabled smart personal devices (*phones, tablets and laptops*) an opportunity is created that can revolutionize, not only the scope, but also the process healthcare. mHealth (or m-health) is an abbreviation for mobile health, a term used for the practice of medicine and public health supported by mobile devices such as: mobile communication devices, mobile phones, tablets, PDAs, and wearable devices such as smart watches, for health services, information, and data collection, information exchange and communication *via* the Internet. mHealth is one aspect of eHealth that is pushing the limits of how to acquire, transport, store, process, and secure the raw and processed data to deliver meaningful results. mHealth started at the industrialized nations but emerged in recent years as an application for developing countries, stemming from the rapid rise of mobile phone penetration in low-income nations where they face a plethora of constraints in their healthcare systems. It provides greater access to larger segments of a population in developing countries and improves the capacity of health systems in such countries to provide quality healthcare. But there are concerns about the accuracy and unregulated status of health apps.

The Internet of Things concept has three pillars for its development:

*Connectivity:* The universalization of Internet allows everyone to connect with high bandwidth through cellphones or wireless networks (WiFi).

*Sensing Devices:* Universalization of cheap devices with sensing capabilities. There are sensors for any of the five human senses. Smartphones can sense in which position they are, if the user is looking at them, if the user is speaking to them, how fast the user is moving and where in the world it is placed. Recently are in development more sophisticated sensors that can detect smells and flavors.

*Computational Power:* Not only today’s devices (like smartphones, tablets) have the same computational power of IBM’s 80s computers of the size of a room, but any device is able to access additional computational power through the Cloud and its virtual services.

The digital divide describes the differences between those who have access to the Internet and those who do not because of economic reasons. It describes also the lack of computer competency or self-efficacy and/or the lack of communications infrastructure. This situation can impact negatively the use of the above described technologies in many areas of our world. This can be partially compensated using specialized applications off-line on computers or mobile devices.

**Ivan Brandslund** (Odense, Denmark) continued the session with a talk on **“AI in cancer, emergency and COVID-19”.** Artificial Intelligence (AI) is a promising technology to use in analysis of numerous data and in diagnostics. Based on the assumption that the effect of any disease would change the pattern of laboratory test results and thus a possibility that analysis of these patterns could evaluate absolute risk for specific diseases, Dr Brandlund et al. have tested the ability of AI to predict risk of cancer in patients consulting their general practitioner, the predictive values for sepsis, death and specific diseases in emergency received patients as well as analyzed consecutive data from patients admitted with severe disease caused by COVID-19.

The results were that the risk score of cancer can be measured from 0% to 80%, emergency-received patients outcome could be predicted with area under the curve in ROC analysis of between 87% and 92% and that the analytical test with the highest predictive value for outcome in COVID-19 is the absolute concentration of virus particles in the blood at admittance. AI is likely to be a part of the clinical diagnostic methodologies within the next 10 years.

### 3.11 Session VIII–pharmacogenomics and post-marketing applications

The session was chaired by **Charity Nofziger, Salzburg, Austria** and **Vangelis Manolopoulos, Alexandropoulis, Greece**

The first speaker of this session, **Ron HN van Schaik** (Rotterdam, Netherlands), talked about **“Pharmacogenetics testing in a healthcare system: opportunities and challenges”.** For the most effective use of pharmacogenetic testing, the uptake in a healthcare system is key. This stretches from easy and straightforward ways to ask for testing, easy sample collection, trustable, accurate and timely results, but also reporting and reimbursement issues. When in a country, several laboratories are performing the testing, harmonization of testing, interpretation and reporting become essential topics. In Netherlands, they managed to implement much of this chain, including that every pharmacist in the country can provide genotype-based dosing advice for more than 100 drugs. To have the system operating, continuous education of specialist, GPs, pharmacists, students, patients and insurers was organized. One challenge still to be addressed in the country is the fact that genotype to phenotype interpretations may change over time. It is important to have genotype portals, or national electronic health records available to notify already genotyped patients about any changes in interpretation. Although this brings another aspect forward: how does the patient react to this change of interpretation when they were treated based on the original reports? This is an interesting challenge that should be discussed within the scientific community.

Next, **Charity Nofziger** (PharmGenetix GmbH, Anif/Niederalm, Austria), presented **“Efforts of the Pharmacogene Variation Consortium (PharmVar) to facilitate the interpretation of pharmacogenetic test results and guide precision medicine”.** The Pharmacogene Variation Consortium (PharmVar) is the home for pharmacogene nomenclature that serves as a centralized data repository for single nucleotide variants (SNVs) in PGx related genes. Its main goal is to catalogue allelic variation in genes that play a role in the metabolism, disposition and response to drugs, and provide a unifying and standardized nomenclature system for the entire PGx community. Dr Nofziger gave a quick overview of the website and highlighted its main features, tools and resources to facilitate PGx related work.

**Ferrier Le** (Thermo Fisher Scientific, Santa Clara, United States) presented a **“Method for *CYP2D6* Copy Number Variation Analysis using Multiplex Digital PCR on the QuantStudio Absolute Q System”.** The team developed a method using multiplex TaqMan^®^ digital PCR to analyze *CYP2D6* copy number variation using the QuantStudio™ Absolute Q™ Digital PCR System. The system’s four optical channels enabled the development of a custom 4-plex digital PCR assay for the copy number variation analysis of three *CYP2D6* regions (exon 9, intron 2, and 5′ UTR) in a single digital PCR reaction. The assay performance was verified using a panel of reference DNA samples. Compared to existing methods, the workflow using the Absolute Q system with the multiplex *CYP2D6* assay reduced the time to results for copy number variation analysis without compromising accuracy or performance.

The session continued with **Ingolf Cascorbi** (Kiel, Germany), talking about “Pharmacogenomics of tyrosine kinase inhibitor resistance”. Tyrosine kinase inhibitors (TKI) are widely used in the modern treatment of malignancies, such as the treatment of chronic myeloid leukemia (CML) through imatinib, inhibiting the catalytic domain of the BCR-ABL fusion gene product. However, resistances may be caused by BCR-ABL independent mechanisms. The group identified mechanisms of drug resistance applying CML *in-vitro* models of imatinib as well as nilotinib resistance. They were able to demonstrate significant upregulation of the ABCG2 efflux transporter with strong association to deregulation of specific miRNA. These mechanisms could be reversed only under treatment-naive conditions. Further investigations revealed substantial changes of gene expression and (epi)genetics, related established oncogene signaling pathways but interestingly also to cell adhesion pathways (Kaehler and Cascorbi, 2021).

The final talk in this session was by **Belgin Süsleyici** (Istanbul**,** Turkey) on “**Precision Medicine in Routine Turkish Clinical Practice: Now and in the Future”.** In recent years, traditional treatment plans have been augmented by precision medicine approaches. However, there are still significant issues to be overcome in incorporating these approaches into routine care and integrating new research data to clinical practice. In collaboration with many clinicians and scientists, and with significant support of the Ministry of Health in Turkey, the group started studies by performing pharmacogenetic analyses according to the drug-use status of patients from cardiology, oncology, psychiatry as well as physical therapy and rehabilitation clinics. The effects of *CYP2C9* and *VKORC1* polymorphisms on warfarin-dose requirements in Turkish patients were determined and as a conclusion to the *CYP2C9* *2, *3, *VKORC1* 9041 G**>**A polymorphisms were found to explain the considerable proportion of inter-individual variability in warfarin dose requirement. The individualized metoprolol doses to be used in treatment of heart rates and blood pressures for cardiac patients and tramadol responses for physical therapy and rehabilitation patients were related to CYP2D6 genotyping, therefore pharmacogenetic results of CYP2C6 are being considered before drug prescriptions. In oncology, the relationships between 5-FU treatment-related adverse events and *DPYD*, *MTHFR* and *TYMS* gene polymorphisms involved in 5-FU metabolism in colorectal cancer (CRC) patients were evaluated. Together with genes affecting 5-FU response, the relationship between bevacizumab use and specific *VEGF* polymorphisms, have been investigated based on the survival time and metastasis in colorectal cancer patients. The results obtained from the *PDL-1* checkpoint inhibitor have been used to produce a real-time PCR kit that is able to help clinicians determine the immunotherapeutics they will be using in targeted therapies. Based on the assumption that the variations in endothelial-mesenchyme transformation inducers like *SNAI1* and *LOXL2*, may have synchronous effect on metastases resulting with malignant phenotype, genotyping results are being recorded for these gene polymorphisms for their clarification to be used in PGx routine. This data will contribute to the understanding of both malignancy and the potential of new therapeutic targets to be used in the treatment processes of various disease.

### 3.12 Posters

Twenty-five posters (**Supplementary)** were presented that were classified in two groups (group 1: “-omics” biomarkers and group 2: Pharmacogenomics). Jaroslav Hubacek and his team won the “Omics” poster award, granted by The Santorini Conferences Association (SCs), for their work on the “Apolipoprotein L1 variability is associated with increased risk of renal failure in the Czech population”. Päivi Hirvensalo and her team won the “Pharmacogenetics” award, granted by the European Society of Pharmacogenomics and Personalised Therapy (ESPT), for their work on “Pharmacogenomics of celiprolol”.

## 4 Conclusion

During the 10th Santorini Conference, we enjoyed high quality talks and poster presentations, covering a range of recent advances in the area of personalized medicine. World-renowned scientists discussed state of the art approaches of introducing PGx in the clinical practice, the use of liquid biopsies in the prognosis of cancer, the use of biomarkers, genetic and polygenic risk scores and multi-omics in the prediction of cardiometabolic traits and the implementation of AI in different areas of research and patients’ treatments. We are looking forward to our next conference scheduled for 21–24 May 2024.
