# Supplementary material for: The 10th Santorini conference: Systems medicine, personalised health and therapy. “The odyssey from hope to practice: Patient first. Keep Ithaca always in your mind”, Santorini, Greece, 23–26 May 2022
Source: Front Genet. 2023 Mar 9;14:1171131. doi: 10.3389/fgene.2023.1171131 (PMC10069673; doi:10.3389/fgene.2023.1171131)
Supplement: Supplementary file 1 [file DataSheet1.DOCX]

# Supplementary

The 10th Santorini Conference: Systems Medicine, Personalised Health and Therapy. “The Odyssey from Hope to Practice: Patient First. *Keep Ithaca always in your mind*”, Santorini, Greece, 23-26 May, 2022

Sophie Visvikis-Siest^1,*,**^, Maria G. Stathopoulou^2,*^, Raute Sunder-Plassmann^3,*^*,* Behrooz Z. Alizadeh^4,*^, Robert Barouki^5^, Ekaterina Chatzaki^6,7^, Georges Dagher^8,9,10,11*^, George Dedoussis^12^, Panagiotis Deloukas^13,*^,  Alexander Haliassos^14^, Brigitte Boisson Hiegel^1,*^, Vangelis Manolopoulos^6,15^, Christine Masson^1^, Guillaume Paré^16,*^, Markus Paulmichl^17^, Alexandros M. Petrelis^1,18^, Csilla Sipeky^19^, Belgin Süsleyici^20,*^, Georges Weryha^1,*^, , Alex Chenchik^21^, Paul Diehl^21^, Robin E. Everts^22^ Alexander Haushofer^23,*^, John Lamont^24,*^ Ruth Mercado^21^, Heiko Meyer^25,*^, Herna Munoz-Galeano^26,*^, Helena Murray^24^, Ferrier Nhat^27^, Charity Nofziger^28,*^, Wolfgang Schnitzel^28^, Stavroula Kanoni^13,*,**^.

^1^ EA_1122; IGE-PCV, Université de Lorraine, Nancy, France;

^2^ Team 10: Control of Gene Expression, INSERM U 1065, Centre Méditerranéen de Médecine Moléculaire C3M, Nice, France;

^3^ Department of Laboratory Medicine, Medical University of Vienna, Währinger Gürtel 18-20, 1090 Vienna, Austria;

^4^ Unit of Personalized Medicine, Department of Epidemiology, University Medical Center Groningen, University of Groningen, Groningen, The Netherland;

^5^ Université de Paris, Inserm unit 1124 (T3S), Paris, France;

^6^ Laboratory of Pharmacology, Medical School, Democritus University of Thrace, Alexandroupolis,Greece

^7^ Institute of Agri-Food and Life Sciences, Hellenic Mediterranean University Research Centre, Heraklion, Crete, Greece;

^8^ Inserm, France;

^9^ Graz Medical University;

^10^ Milano-Bicocca University;

^11^ Beijing Academy of Sciences;

^12^ Department of Nutrition and Dietetics, Harokopio University of Athens, Athens, Greece;

^13^ William Harvey Research Institute, Barts and the London School of Medicine and Dentistry, Queen Mary University of London, London, United Kingdom;

^14^ EurSpLM, ESEAP, the Greek Proficiency Testing Scheme for Clinical Laboratories Athens, Greece;

^15^ Clinical Pharmacology & Pharmacogenetics Unit, Academic General Hospital of Alexandroupolis, Alexandroupolis, Greece;

^16^ Population Health Research Institute, Genetic and Molecular Epidemiology Laboratory, McMaster University, Hamilton, Ontario, Canada;

^17^ Privatklinik Maria Hilf, Klagenfurt, Austria;

## ^18^ Digitsole, Nancy, France;

## ^19^ UCB Pharma, Translational Medicine, Precision Medicine & Biomarkers, Genetics, Braine-l’Alleud, Belgium;

^20^ Marmara University, Faculty of Sciences and Letters, Department of Molecular Biology, Istanbul, Turkey;

^21^ Cellecta, Inc., Mountain View, California, USA;

^22^ Agena Bioscience, San Diego, CA, USA;

^23^ Inst. f. Med. u. Chem. Labordiagnostik, Klinikum Wels-Grieskirchen GmbH, Wels Austria;

^24^ Randox Laboratories Limited, Crumlin, Co. Antrim, United Kingdom;

^25^ Agena Bioscience, Hamburg, Germany;

^26^ HMG systems engineering GmbH, Fürth, Germany;

^27^ Thermo Fisher Scientific, San Francisco, California, United States;

^28^ PharmGenetix GmbH, Anif/Niederalm, Austria;

^*^ The Santorini Conferences Association (SCs), Bernecourt, France.

^*^ ^*^ Corresponding authors.

**FLASH COMMUNICATIONS SESSION**

The session was chaired by **Stavroula Kanoni, London, United Kingdom** and **Vangelis Manolopoulos**, **Alexandroupolis, Greece**

ICLE Open Access

**Janja Marc (**Ljubljana, Slovenia), started the session with a talk on **“How men have stronger bones - Sry regulation of *RANKL* expression”**. Receptor activator of nuclear factor κB ligand (RANKL) is one of major regulator of osteoclasts activity and bone resorption. Factors regulating *RANKL* expression may therefore indirectly influence bone quality and development of bone diseases. The frequency of osteoporotic bone fracture in men is about half as many fractures as women, at any given age (Adler, 2014). The research team aimed to find the gender specific regulators of *RANKL*. They employed gene transfection for overexpression of Sry and silencing of Sry by siRNA in human primary osteoblasts followed by luciferase reporter assays and electrophoretic mobility shift assays. A total of 112 human bone samples were examined using immunohistochemical examination and qPCR. The research team discovered that transcription factor, sex-determining region Y (SRY), regulate *RANKL* expression and demonstrated that male-specific SRY decreases *RANKL* expression through direct binding to its 5’-proximal promoter. These results were corroborated by the gene expression in human bone samples. In osteoporotic men, expression of *RANKL* is 17-fold higher, which correlates with the drastically reduced expression (200-fold) of Sry, suggesting that in osteoporotic men, the upregulation of *RANKL* is caused by a decrease of Sry. In healthy men, the expression of *RANKL* is 20% higher than that in healthy women. This data suggests that gender differences in *RANKL* expression and bone quality could be due to the sex-specific transcription factor SRY (Kodric et al., 2019).

Next, **Milica Medić Stojanoska** (Novi Sad, Serbia) presented their recent work on **“Polycystic ovary syndrome: do phthalates and bisphenol A play role?”**. The widespread use of plastics made phthalates and bisphenol A (BPA) omnipresent in the environment. The frequent detection of these endocrine disrupting chemicals during biomonitoring studies in the general population are related to greater incidence of obesity, dyslipidemia, insulin resistance and impaired thyroid function which are elements of polycystic ovary syndrome (PCOS). The aim of this cross-sectional study was to determine if plastic chemical exposures can contribute to reproductive disorders such as PCOS. The concentration levels of phthalate metabolites and BPA in morning spot urine of women in reproductive period were determined by gas-chromatography coupled with mass spectrometry. Based on the results presented, half of the examined PCOS women had BPA (48.3%) and phthalate metabolites (51.3%) in their urine. The presence of BPA in urine of PCOS women were associated with insulin serum levels insulin serum levels (*p*=0.038), elevated HOMA-IR values (*p*=0.079) and reduced HDL (*p*=0.061). Also, there was almost 4 times higher risk for PCOS BPA+ women to have testosterone levels above reference values. Concentration levels of sum of all phthalates metabolites significantly correlated with total cholesterol (*p*=0.028) and LDL (*p*=0.07). These results indicate that exposition to BPA and some types of phthalates in PCOS women lead to the increased metabolic risk (Stojanoska et al., 2017) and consequently may aggravate PCOS clinical complications. This work was supported by the Provincial Secretariat for Science and Technological Development, AP Vojvodina, Republic of Serbia (Grant No. 114-451-2394/2011; Grant No. 114-451-2216/2016).

Following this talk, **Sanja Stankovic** (Belgrade, Serbia), presented their work on the: **“Association between CYP2C19 polymorphisms and clinical outcomes in STEMI patients treated by PPCI”**. Primary percutaneous coronary intervention (PPCI) is nowadays the preferred reperfusion strategy for treating ST-elevation myocardial infarction (STEMI) patients, aiming at restoring epicardial infarct-related artery patency and achieving microvascular reperfusion as early as possible. Clopidogrel is a commonly used antiplatelet drug to reduce the rate of procedure-related thrombosis. The response to clopidogrel varies widely among individuals. Differing responses to clopidogrel may be related to CYP2C19 gene polymorphisms. Although the relationship between CYP2C19*2/CYP2C19*3 alleles and increased incidence of stent thrombosis is well known, CYP2C19*17 impact on clinical outcome is an area that requires more investigation. This study aimed to investigate the clinical impact of CYP2C19 gene polymorphisms in STEMI patients (140) treated by PPCI, with the use of Multiple Electrode Aggregometry. The primary end-points were major adverse cardiovascular/cerebrovascular events (MACE/MACCE) and major bleeding. The results suggested an increased risk of ischemic events following PPCI in carriers of CYP2C19*17, independent of clopidogrel responsiveness.

The next speaker was **Adrián Llerena** (Badajoz, Spain) and presented: **“MedeA – Development of a Clinical Support System for PGx clinical Implementation based of e-health**”. The MedeA Initiative is a genuine personalized medicine (PM) clinical implementation programme. It is based on an analysis of each patient’s individual pharmacogenetic biomarkers, drug-drug interactions and clinical data evaluated in an e-health strategy. This information is fed into a software system called Personalized oriented Prescription System (PoPS) integrated within the Electronic Medical Record (EMR) system. The aim is to develop an individualised drug prescription decision supporting tool for regular clinical practice by assessing and analysing pharmacogenetic information and other variables (clinical data and drug prescription) from the EMR with artificial intelligence algorithms. The project is implemented for all patients of the Public Health System in the Spanish region of Extremadura. This initiative would be applicable to any health care setting developing an automated personalised prescription system promoting drug safety and efficacy. It is conceptualized as a proof of concept for PM implementation and could be taken up by other European health care services, contributing to their sustainability and improving the treatment of patients.

The last speaker of this session was **Vangelis Manolopoulos** (Alexandroupolis, Greece), presenting: **“Pharmacogenomics and Personalized Medicine in Anticoagulation: latest developments”**. In recent years, the therapeutic arsenal for anticoagulation has gradually grown and currently includes, in addition to the older coumarinic anticoagulants (COAs), four novel compounds, the direct thrombin inhibitor dabigatran, and the factor X inhibitors rivaroxaban, apixaban, and edoxaban, collectively referred to as direct oral anticoagulants (DOACs). Application of COAs PGx is useful both in predicting the optimal doses of COAs and in identifying individuals who are at increased risk of bleeding with COAs and, consequently, would benefit with a DOAC rather than COAs. Regarding DOAC PGx, so far there have been only sparse data, most notably a GWAS in patients treated with dabigatran, and only a few polymorphic genes have been studied in association with DOAC response. In addition, some answers in relation to the heterogeneity in patient responses to both these classes of drugs may be found at the epigenetic level, through the clinical study “Epigenetic modifications (DNA methylation and miRNAs) as regulators of response to DOACs in atrial fibrillation (The MiR-CRAFT study)”

**SELECTED ABSTRACTS - ORAL COMMUNICATIONS SESSION**

Session chaired by **Stavroula Kanoni, London, United Kingdom** and **Vesna Dimitrejevic Sreckovic, Belgrade, Serbia**

**The first speaker of this session, Didier Bourgeois (**Neuilly sur Seine, France), presented **“****A Prospective Multicenter French Study of the 21-Gene Assays: Impact on Treatment Selection in Patients with High-Risk Breast Cancer”.** The 21-gene Oncotype DX Recurrence Score has been extensively validated to predict the risk of distant recurrence and the magnitude of response to hormone and chemotherapy in patients with ER+ N0 and N1-3+ HER2- early stage breast cancer. The test is available in France via the French public funds program (RiHN) and a market access program is in place that prospectively collects patient information including traditional clinical and pathological features (histology, tumor grade, size, ER, PR, HER2 status and % of Ki67), patient age as well as the 21-gene Recurrence Score Result and treatment recommendations before testing and final therapy prescribed after Recurrence Score results are known. For this study, data was available in 786 patients of which 148 had high-grade (G3) tumors and 249 patients had a high KI67 (>20%). In this cohort 122 (87%) and 159 (75%) of patients with G3 and high KI67 features respectively, had pre-ODX treatment recommendations for chemo-hormonotherapy (CT-HT), that decreased to 82 (58%) and 95 (45%), respectively, post-ODX testing. Post-testing physicians changed treatment decision in 35% and 40% of patients with G3 and high KI67 disease leading to a 28% and 30% net reduction in CT respectively. The 21-gene test provides critical information that changes and supports final treatment decisions in breast cancer patients identified as high-risk according to traditional tumor grade and high Ki67 levels.

The second speaker, **Frances T Yen (Vandoeuvre les Nancy, France)** talked about **“Molecular mechanisms underlying dyslipidemias as risk factors for Alzheimer’s disease”.** Dyslipidemias are established risk factors for cardiovascular disease, and recent evidence reveal an important impact of age-related cognitive decline and Alzheimer’s disease (AD). However, underlying molecular mechanisms remain unclear. A significant clue is provided by apolipoprotein (apo)E; the three allelic variants of APOE have been linked to type III hyperlipidemia (ε2), high plasma cholesterol levels and AD risk (ε4). Lipoprotein receptors involved in regulating apoE-lipoprotein trafficking could therefore represent interesting therapeutic targets for treatment of dyslipidemias and AD. The human LSR gene (coding for the lipolysis stimulated lipoprotein receptor) is located upstream of the APOE gene, and results reveal epistatic interactions between LSR and APOE that can influence blood lipid levels and AD risk in human subjects (Xie et al., 2018). Heterozygote lsr+/- mice demonstrate moderate hypercholesterolemia and hypertriglyceridemia, due to a decrease in hepatic removal of lipoproteins and increased body fat mass (Yen et al., 2008). Furthemore, transcriptomic analysis reveals that these animals have a predisposition to obesity (Akbar et al., 2016). lsr+/- mice reveal age-related changes in brain cholesterol distribution, and increased memory deficits following amyloid stress correlated with cortical cholesterol levels (Pincon et al., 2015). These results suggest that the LSR pathway may represent an important molecular link between dyslipidemias, age-related cognitive decline and AD. Further investigation may reveal reliable lipid biomarkers with predictive values for AD allowing timely intervention for reducing AD risk.

The next speaker was **Lena Neufeld** (Tel Aviv, Israel), describing their **“Perfusable 3D-bioprinted glioblastoma model for in-vivo mimicry of** **tumor microenvironment and personalized medicine****”.** Many drugs and chemical compounds show promising results in laboratory research, but eventually fail clinical trials, mostly due to inadequate cancer models. Most models lack the tumor-matrix interactions, which are essential for proper representation of cancer complexity. **Lena et al.** recapitulated the tumor heterogenic microenvironment by creating a 3D-bioprinted glioblastoma (GB) model, a library of synthetic, natural and chemically modified polymers which were used as bioinks (Neufeld et al., 2021). By studying the interactions between each chemical component on the crosslinking kinetics, the mechanical strength of the printed structure and many other properties to match the brain tissue characteristics, they observed similar growth curves, drug response and genetic signature of GB cells grown in the 3D-bioink platform and in orthotopic mouse models of cancer as opposed to 2D culture on rigid plastic plates.The 3D-bioprinted model could be the basis for potentially replacing cell cultures and animal models as a powerful platform for rapid, reproducible and robust target discovery tool, personalized therapy screening and drug development. The Satchi-Fainaro laboratory’s research leading to these results has received funding from Morris Kahn Foundation.

**The next talk was from Wolfgang Schnitzel (**PharmGenetix GmbH, Niederalm, Austria)**, presenting “New ways of implementing PGx to clinical practice with an interactive web application”.** The effects and tolerability of medications and their active ingredients can vary greatly from person to person. A pharmacogenetic analysis provides important information about the personalized use of medications. However, physicians are usually not trained in pharmacogenetics and therefore it’s challenging to translate PGx data into tailored dose adaptions in the medical practice. Furthermore, PGx-based drug-drug interactions play a key role in drug metabolism and should therefore also be considered for a solid personalized drug prescription. **Dr. Schnitzel** presented an in-house developed web application (PGx-Optimizer) which illustrates the results of a PGx analysis in an easy and actionable way, offering information on recommended drug dosages, as well as possible drug-drug interactions. The objective was to show that a digital tool like the PGx-Optimizer can allow physicians to understand the consequences of a PGx report, reduces the physician’s spent time on the report’s interpretation and finally facilitate the implementation of PGx into daily routine. The results of PGx analyses were provided to hospital-based and office-based physicians via the PGx Optimizer. The physicians were given a crash course on PGx and on the functionality of the program and also received support for the interpretation of the original PGx report. Most of the users rated the PGx-Optimizer as an excellent tool to implement pharmacogenetics. Many physicians reported a noticeable clinical impact for specific patient cases in the context of reduced drug side effects after PGx-guided dose adjustments. For a broader acceptance and usage of pharmacogenetics in daily medical routine, a digital tool providing clear and actionable recommendations is key. The PGx-Optimizer proved its clinical usability and could therefore serve as a door opener for broader PGx implementation.

The next talk was from **Akin Demet** (Istanbul, Turkey) on the **“****Evaluation of Carboxylesterase-1 and Latrophilin-3 Gene Polymorphisms for Methylphenidate Treatment Prediction in Childhood Attention Deficit Hyperactivity Disorder in Turkish Population”.** Attention deficit/hyperactivity disorder (ADHD) is a childhood-onset behavioral and neurodevelopmental disorder (NDD), affecting 5% of children worldwide. It is characterized by inattention, hyperactivity, and impulsivity. The precise cause or causes are unknown in most cases; however, genetic factors are estimated to make up about 75% of the etiology of ADHD, mostly in regulatory regions. Besides genetic factors it is anticipated that environmental risk factors likely work through epigenetic mechanisms. Psychostimulants are the first-line pharmacotherapy for ADHD. Methylphenidate (MPH) accounts for approximately 50% of all stimulant usage worldwide in childhood ADHD that exerts its therapeutic effect by blocking dopamine transporters (DAT) and norepinephrine transporters (NET). However, there is a considerable interindividual variability exists in MPH treatment. Up to 35% of ADHD patients do not respond satisfactorily to MPH therapy, and a significant number of patients suffer from different adverse effects such as, decreased appetite, nausea, headache, insomnia causing to treatment discontinuous. It is reported that polymorphisms in carboxylesterase 1 (CES1) gene and in latrophilin 3 (LPHN3) are associated with response to MPH. In this study, the researchers aimed to evaluate the association between MPH therapeutic outcomes in childhood ADHD response and genetic variants of CES1 and LPHN3 in Turkish population by revealing key associations between CES1 and LPHN3 genotypes and PK and PD of MPH, respectively. This study (Elsayed et al., 2020) included 200 patients (70% boys), aged 6 to 17 years, newly diagnosed (medication-naïve) with ADHD according to the DSM-5.

**Reem Hamad** (Khartoum, Sudan) presented **“Clinical exome sequencing in the African setting: challenges and opportunities”.** Africa's socio- economic conditions, meager health resources and inefficient health system is perhaps not the ideal situation to foster adoption of new genomic technologies but its spectacular genetic variation might render it imperative to implement such approaches for better clinical outcomes. In this study (Koko et al., 2018), exome and whole genome sequencing were adopted both in research and in clinical diagnostic settings to investigate various familial conditions and genetic abnormalities. The results revealed disparate and complex etiologies some of unusual genotype phenotype relationships. This relationship reflects the continent legacy of genetic and epigenetic heterogeneity and complexity. There must be a change in our perspective on diagnosing diseases and genetic disorders in Africa building on its population genetic heterogeneity and embarking on serious investments in public health genomics.

In the final talk for this session, **Balazs Gyorffy (**Budapest, Hungary) described **“A machine learning method estimating the benefit of adjuvant therapy in breast cancer”.** Different treatment regimens can improve outcome in breast cancer. However, in multiple instances there is no established protocol for the selection of the most suitable treatment for an individual patient. The researchers created a model that provides personalized survival and treatment response estimation based on available clinical and pathological parameters for a breast cancer patient. Data was integrated from multiple repositories and databases (TCGA-CDR, SEER, METABRIC, IMPACT, GSE96058). K-means clustering analysis was employed to identify clinically relevant clusters. Multi-task logistic regression model was trained within the group of most similar cases identified by machine learning SVM for the case under investigation. Survival estimations were visualized by the Kaplan-Meier method, and significance was calculated by a log-rank test. The discriminative ability and the calibration of the individual survival prediction model was validated by the C-index, and the 1-calibration methods, respectively. Clinical data from all together 354,172 breast cancer cases were incorporated, and 51,206 cases from these had complete follow-up and treatment data. Twelve clusters with characteristic outcome and therapeutic response were identified. New cases were assigned into the closest cluster with an accuracy over 97.7%. The prediction model was well calibrated for 2-6 years from diagnosis at p<0.01, overestimating 5-year overall survival by 1.81%. The C-index values for the model’s discriminative capacity were 0.77 and 0.76 for five- and ten-year overall survival, respectively. The authors concluded that they have constructed a well-calibrated artificial-intelligence based survival prediction model suitable for different cohorts of breast cancer patients.

**POSTERS**

**GROUP A – “-Omics”**

**Alex Chenchik** in the poster entitled **“Cell Barcoding, Genetic Screens and Expression Profiling at a Single-cell Level”** presented a panel of lentiviral barcoded sgRNA libraries developed to label and monitor cancer cells in time course experiments in vitro and in mouse xenograft models. Data were presented concerning the applications of a genetic screen technology combined with targeted RNA expression profiling of barcoded knockout cells that can improve phenotyping of distinct cell populations in several cancer model systems. The assay has significant throughput, sensitivity, and improved cost-effectiveness allowing applications on high-throughput drug target discovery.

**Alexandros Petrelis** presented a poster entitled **“Associations of VEGF-A levels with inflammatory molecules”**. Significant associations were identified between inflammation molecules and VEGF-A levels and gene expression isoforms in PBMCs extracts, thus providing novel insights that may assist in the development of tissue and mRNA isoform specific measurements of VEGF-A levels. These could positively contribute to predicting the risk of common complex diseases and response of currently used anti-VEGF-A agents and developing of novel targeted therapies for VEGF-A related pathophysiology.

**Alexandros Petrelis** also presented the poster **“Gait alterations in Diabetic patients without peripheral neuropathy in comparison to healthy individuals”**. In a case-control study of type 2 diabetes, patients without clinical signs of peripheral neuropathy nor diabetes distress, have significant differences in several gait parameters, thus proposing that the investigation of gait analysis could be an early diagnostic tool of diabetes complications.

**Frances Yen** in the poster **“Regulation of brain cholesterol trafficking by lipolysis stimulated lipoprotein receptor in murine astrocytes – potential role in Alzheimer’s disease”**, demonstrated that 75% LSR knockdown in murine astrocytes increased the expression of enzymes involved in cholesterol synthesis and secretion, while significant decrease in cell cholesterol content and a 2-fold increased media apoE supported the notion of increased cholesterol efflux following knockdown of LSR expression. These results suggested that LSR can act as lipid sensor and at low levels, can drive cholesterol flux extracellularly towards the neuron.

**Ioanna-Panagiota Kalafati** in the poster entitled **“Physical activity alters the genetic predisposition of non-alcoholic fatty liver disease (NAFLD) in a Greek case-control study”** demonstrated the development of a weighted genetic risk score (wGRS) and tested it for associations and interactions with physical activity on NAFLD odds.
The results suggested that physical activity can modify the genetic predisposition to NAFLD.

**Lena Neufeld** in the poster entitled **“Higher similarities in the transcriptional profile between in-vivo and 3D-bioprinted cancer models compared to 2D cultures”** presented the creation of a 3D-bioprinted glioblastoma (GB) model andevaluated the transcriptome of GB cells grown in 2D, in the 3D-bioink or isolated from GB tumors in mice. The cells grown in the fibrin 3D-bioink were more similar to the cells isolated from mice brain than the 2D culture samples. Analysis of differentially expressed genes between the 3D and the 2D culture identified 6936 differentially expressed genes in pathways including proliferation, cell-cell interaction, adhesion, inflammatory response, angiogenesis and several oncogenic markers. Thus, expressed genes in fibrin 3D-bioink provided essential cues to enhanced tumor growth and survival, that were inefficiently expressed in standard 2D culture.

**Ekaterini Chatzaki** presented the poster with the title **“Liquid biopsy diagnostics in type 2 diabetes mellitus”** that was focused on the study of circulating cell-free DNA (ccfDNA) as a liquid biopsy biomaterial aiming to build diagnostic/monitoring predictive biosignatures for clinical application.

The same team presented the poster with the title **“Disease-specific biosignatures via automated machine learning methylome analysis”**. An in-silico pipeline based on high-throughput methylome datasets was used to identify disease/tissue specific methylation patterns. Three pathological entities of major burden, i.e. Breast Cancer (BrCa), Osteoarthritis (OA) and Diabetes were used as case studies. Several Differential Methylated Genes (DMGs) emerged automated machine learning delivered a 5-gene biosignature discriminating BrCa tissue against healthy tissues, 3 equivalent OA cartilage specific biosignatures and a 4-gene pancreatic β-cell specific biosignature. Thus, with the use of an innovative analytical tool, simple and highly performing methylation biosignatures for clinical implementation were produced.

**Maja Milanović** in the poster entitled **“Preliminary results of phthalates exposure in children and young adults”** described that in a study of 103 children and young adults, phthalates above limit of quantification were found in 8.7% of the participants in this study. MEP was detected with higher frequency in young women (87.5%).

**Jaroslav A. Hubáček** in the poster entitled **“Genetic risk score as a potential predictor of T2DM in Czech population”** presented the associations of selected variants in genes for *FTO*, *CDKN2A/2B*, *THADA*, *IRS1*, *IGF2BP2*, *MAEA*, *JAZF1*, *CDKAL1*, *TCF7L2*, *ARAP1*, *TLE4*, *ADCY5*, *ZMIZ1* and *KCNJ11* in 855 T2DM patients and 2339 adult controls. The associations of some but not all of the analyzed variants with the development of T2DM was confirmed indicating that different genes may be associated with this phenotype in different populations.

## The same team in the award-winning poster entitled “Apolipoprotein L1 variability is associated with increased risk of renal failure in the Czech population” presented the associations of six APOL1 SNPs (rs73885319, rs71785313, rs13056427, rs136147, rs10854688 and rs9610473) and one newly detected 55-nucleotide insertion/deletion polymorphism with end stage chronic kidney disease (1,489 kidney transplantation patients and 2,559 healthy controls). An association between rs10854688 and end-stage renal disease in the Czech Caucasian population was found while prevalence of the newly detected 55-bp APOL1 deletion was significantly higher in patients.

**Raad Aris Besharat** in the poster entitled **“Identification of molecular signatures in advanced epithelial ovarian cancer patients by liquid biopsy: a pilot study”** demonstrated 431 differentially expressed (DE) miRNAs in plasma and 163 DE miRNAs in urine samples between patients with ovarian cancer and controls, revealing 138 circulating miRNAs combined that could be of value as diagnostic biomarkers. Comparison of the plasma, urine and plasma before and after surgery DE miRNA profiles highlighted 10 circulating miRNAs and among these, miR-221-3p, miR-369-3p and miR-425-3p were up-regulated, being of particular interest since other studies have reported their expression in ovarian cancer.

**Anna Papadopoulou** in the poster entitled **“Molecular and Clinical Profile of patients referred as Noonan or Noonan-like Syndrome in Greece: a cohort of 86 patients”** presented the results of Sanger and Next-Generation-Sequencing of 14 different genes of a Greek cohort of 86 Noonan syndrome (NS) or NS-like patients. Craniofacial dysmorphism, neurological defects and pulmonary valve stenosis prevail among mutation positive compared to mutation negative patients. The significant prevalence of the Ras/MAPK
mutations, as well as the successful treatment of NS patients with RIT1 or SOS1 mutation with selective reversible allosteric inhibitor of MEK1/2 activity reported recently, highlight the necessity of molecular diagnosis in NS patients.

**Georgia Kapoula** presented the poster entitled **“HE4, a new potential useful biomarker, in breast, colorectal and pancreatic cancer patients A primary study, systematic review and meta-analysis”**. In 40 healthy and 60 metastatic patients with breast, colorectal and pancreatic cancer serum human epididymis protein 4 (HE4) was measured and demonstrated a good discriminating value between controls and patients with various cancers and could be used as a good potential diagnostic biomarker.

**Vesna Dimitrijevic Sreckovic** in the poster entitled **“SiMS score in normal weight and overweight/obese women with polycystic ovary syndrome”** aimed to compare the siMS score in normal weight and overweight/obese women with polycystic ovary syndrome (PCOS) and demonstrated that the SiMS score in overweight/obese women is statistically significantly higher than in normal weight women with PCOS. Correlations of siMS score with glycoregulatory and liver parameters indicate the risk of glycoregulation disorders and non-alcoholic fatty liver disease in overweight and obese women with PCOS in conditions of hyperinsulinism and increased IR.

The same team in the poster entitled **“Examining the correlation between acne and parameters of metabolic syndrome, polycystic ovary syndrome and vitamin D values”** aimed to examine of the influence of metabolic syndrome and PCOS parameters, along with vitamin D on the development of acne. Significant correlations were found between the presence of acne and the parameters of the metabolic syndrome - primarily signs of insulin resistance, sex hormones in PCOS, as well as vitamin D deficiency.

**GROUP B –** “**Pharmacogenomics**”

**Salvador Sara** in the poster entitled **“Whole transcription profile of responders to anti-TNF drugs in pediatric inflammatory bowel disease”** identified genes that were differentially expressed in non-responders and responders of anti-TNF treatment for pediatric inflammatory bowel disease (IBD). After validation, FCGR1A, FCGR1B, and GBP1 were overexpressed in non-responders 2 weeks after initiation of the treatment, thus they could be used as pharmacogenomic biomarkers of early response to anti-TNF agents in pediatric IBD.

**Feng Deng** in the poster entitled **“Toxicity and Therapy Outcome Associations in High-grade Serous Ovarian Cancer’’** investigated the genetic association between selected candidate polymorphisms and chemotherapy-induced toxicity and therapeutic outcome in a Finnish high-grade serous ovarian cancer (HGSOC) cohort. LIG3 and SLCO1B3 variants were found to increase the risk of adverse effects, and the GSTP1 variant to affect the first-line treatment response. Moreover, ABCB1 and OPRM1 variants may have an impact on prognosis.

**Johanna Kiiski** presented the poster with the title **“Solanidine is a highly sensitive and specific biomarker for CYP2D6 activity”** that aimed to identify possible biomarkers present in human plasma for measuring CYP2D6 activity through metabolomics analysis in the fasting plasma samples from 355 healthy volunteers. The potato glycoalcaloid solanidine was found to be a highly promising, sensitive, and specific biomarker for measuring CYP2D6 activity.

**Minna Lehtisalo** in the poster entitled **“A comprehensive pharmacogenomic study indicates roles for *SLCO1B1*, *ABCG2*, and *SLCO2B1* in rosuvastatin pharmacokinetics”** investigated the effects of genetic variability on rosuvastatin pharmacokinetics in 247 healthy volunteers. The *SLCO1B1*, *ABCG2*, and *SLCO2B1* genotypes were found to affect rosuvastatin pharmacokinetics possibly through changes in rosuvastatin bioavailability (ABCG2, SLCO2B1) and hepatic uptake of rosuvastatin (SLCO1B1).

In the award-winning poster entitled **“Pharmacogenomics of celiprolol”**, **Päivi Hirvensalo** aimed to study the effect of variations in pharmacokinetic genes on celiprolol exposure in 195 healthy volunteers. Genetic variants in *ABCB1*, which encodes P-glycoprotein, and *SLCO1A2*, which encodes organic anion transporting polypeptide 1A2, were found to be associated with celiprolol pharmacokinetics. Moreover, individuals carrying the *ABCB1* c.2677A or the *SLCO1A2* c.516C allele together with the *ABCB1* c.3435C allele may have a risk of low celiprolol exposure and may thus be at an increased risk of poor blood pressure-lowering efficacy of celiprolol.

**Denis Guyotat** presented the poster entitled **“Predicting treatment free remission in chronic myeloid leukemia : ôle of bcr-abl transcript type”** and showed that in 210 patients with chronic myeloid leukemia (CML) initially treated with imatinib (158 patients) and second generation TKI (46 patients), the type of bcr-abl transcript at diagnosis was predictive of sustained TFR, and may be used to tailor the time to stop treatment.

**Freya Vaeyens** in the poster entitled **“Comprehensive somatic testing of KRAS-G12C-positive colorectal cancers enhances personal treatment selection of patients”** performed a comprehensive testing in Kirsten Rat Sarcoma virus (KRAS)-G12C-positive non-small-cell lung cancer (NSCLC) (n=457) and colorectal cancer (CRC) (n=405) patients in order to identify possible targetable co-drivers to combat intrinsic resistance mechanisms to KRAS-G12C-inhibitors. They demonstrated that somatic testing of KRAS-G12C-positive CRC is of substantial benefit in CRC-treatment decision-making as it is creating the possibility to develop and implement effective combination therapy regimens to combat resistance mechanisms that emerge during treatment with adagrasib and sotorasib.

Finally, **Theodosia Charitou** in the poster entitled **“Drug genetic associations with COVID-19 manifestations: a data mining and network biology approach”** studied the identification of the genomic determinants that influence COVID-19 susceptibility, using a computational, statistical, and network biology approach to analyze relationships of ineffective concomitant medication with an adverse effect on patients. A pharmacogenetic/biomarker network with significant drug-gene interactions originating from gene-disease associations was constructed suggesting that these genes could play a significant role in COVID-19 clinical manifestation due to their association with autoimmune, metabolic, neurological, cardiovascular, and degenerative disorders.

**References**

Adler, R.A. (2014). Osteoporosis in men: a review. *Bone Res* 2**,** 14001. doi: 10.1038/boneres.2014.1.

Akbar, S., Pincon, A., Lanhers, M.C., Claudepierre, T., Corbier, C., Gregory-Pauron, L., et al. (2016). Expression profile of hepatic genes related to lipid homeostasis in LSR heterozygous mice contributes to their increased response to high-fat diet. *Physiol Genomics* 48(12)**,** 928-935. doi: 10.1152/physiolgenomics.00077.2016.

Elsayed, N.A., Yamamoto, K.M., and Froehlich, T.E. (2020). Genetic Influence on Efficacy of Pharmacotherapy for Pediatric Attention-Deficit/Hyperactivity Disorder: Overview and Current Status of Research. *CNS Drugs* 34(4)**,** 389-414. doi: 10.1007/s40263-020-00702-y.

Kodric, K., Zupan, J., Kranjc, T., Komadina, R., Mlakar, V., Marc, J., et al. (2019). Sex-determining region Y (SRY) attributes to gender differences in RANKL expression and incidence of osteoporosis. *Exp Mol Med* 51(8)**,** 1-16. doi: 10.1038/s12276-019-0294-3.

Koko, M., Abdallah, M.O.E., Amin, M., and Ibrahim, M. (2018). Challenges imposed by minor reference alleles on the identification and reporting of clinical variants from exome data. *BMC Genomics* 19(1)**,** 46. doi: 10.1186/s12864-018-4433-3.

Neufeld, L., Yeini, E., Reisman, N., Shtilerman, Y., Ben-Shushan, D., Pozzi, S., et al. (2021). Microengineered perfusable 3D-bioprinted glioblastoma model for in vivo mimicry of tumor microenvironment. *Sci Adv* 7(34). doi: 10.1126/sciadv.abi9119.

Pincon, A., Thomas, M.H., Huguet, M., Allouche, A., Colin, J.C., Georges, A., et al. (2015). Increased susceptibility of dyslipidemic LSR+/- mice to amyloid stress is associated with changes in cortical cholesterol levels. *J Alzheimers Dis* 45(1)**,** 195-204. doi: 10.3233/JAD-142127.

Stojanoska, M.M., Milosevic, N., Milic, N., and Abenavoli, L. (2017). The influence of phthalates and bisphenol A on the obesity development and glucose metabolism disorders. *Endocrine* 55(3)**,** 666-681. doi: 10.1007/s12020-016-1158-4.

Xie, T., Akbar, S., Stathopoulou, M.G., Oster, T., Masson, C., Yen, F.T., et al. (2018). Epistatic interaction of apolipoprotein E and lipolysis-stimulated lipoprotein receptor genetic variants is associated with Alzheimer's disease. *Neurobiol Aging* 69**,** 292 e291-292 e295. doi: 10.1016/j.neurobiolaging.2018.04.013.

Yen, F.T., Roitel, O., Bonnard, L., Notet, V., Pratte, D., Stenger, C., et al. (2008). Lipolysis stimulated lipoprotein receptor: a novel molecular link between hyperlipidemia, weight gain, and atherosclerosis in mice. *J Biol Chem* 283(37)**,** 25650-25659. doi: 10.1074/jbc.M801027200.
